# Who Discovered the Anæsthetic Properties of Ether

**Published:** 1883-06

**Authors:** 


### Who Discovered the Anæsthetic Properties of Ether.

*Gailard's Medical Journal* says that Governor Stephens, of Georgia, made the proposition to place in the National Statuary Gallery at Wasnington the statue of Dr. Long, of Athens, Georgia, who was, said Governor Stephens, "two years ahead of Wells and Morton, in his application of sulphuric ether for the relief of pain in surgical operations, and to whom, therefore, belongs the honor and glory of this greatest discovery of modern times." Dr. Long's claims as a discoverer were strongly advocated by Dr. J. Marion Sims some years since.

